# Nuclear shape is affected differentially by loss of lamin A, lamin C, or both lamin A and C

**DOI:** 10.17912/micropub.biology.001103

**Published:** 2024-02-16

**Authors:** Mai Pho, Yasmin Berrada, Aachal Gunda, Andrew D Stephens

**Affiliations:** 1 Biology Department, University of Massachusetts Amherst, Amherst Center, Massachusetts, United States; 2 Molecular and Cellular Biology, University of Massachusetts Amherst, Amherst Center, Massachusetts, United States

## Abstract

Lamin intermediate filaments form a peripheral meshwork to support nuclear shape and function. Knockout of the LMNA gene that encodes for both lamin A and C results in an abnormally shaped nucleus. To determine the relative contribution of lamin A and C to nuclear shape, we measured nuclear blebbing and circular deviation in separate lamin A and lamin C knockdown and LMNA-/- stable cells. Lamin A knockdown increased nuclear blebbing while loss of lamin A, C, or both increased circular deviation. Overall, loss of lamin A, lamin C or both lamin A/C affect nuclear shape differentially.

**Figure 1. Nuclear blebbing and circular deviation upon loss of lamin A, C, and A/C.**
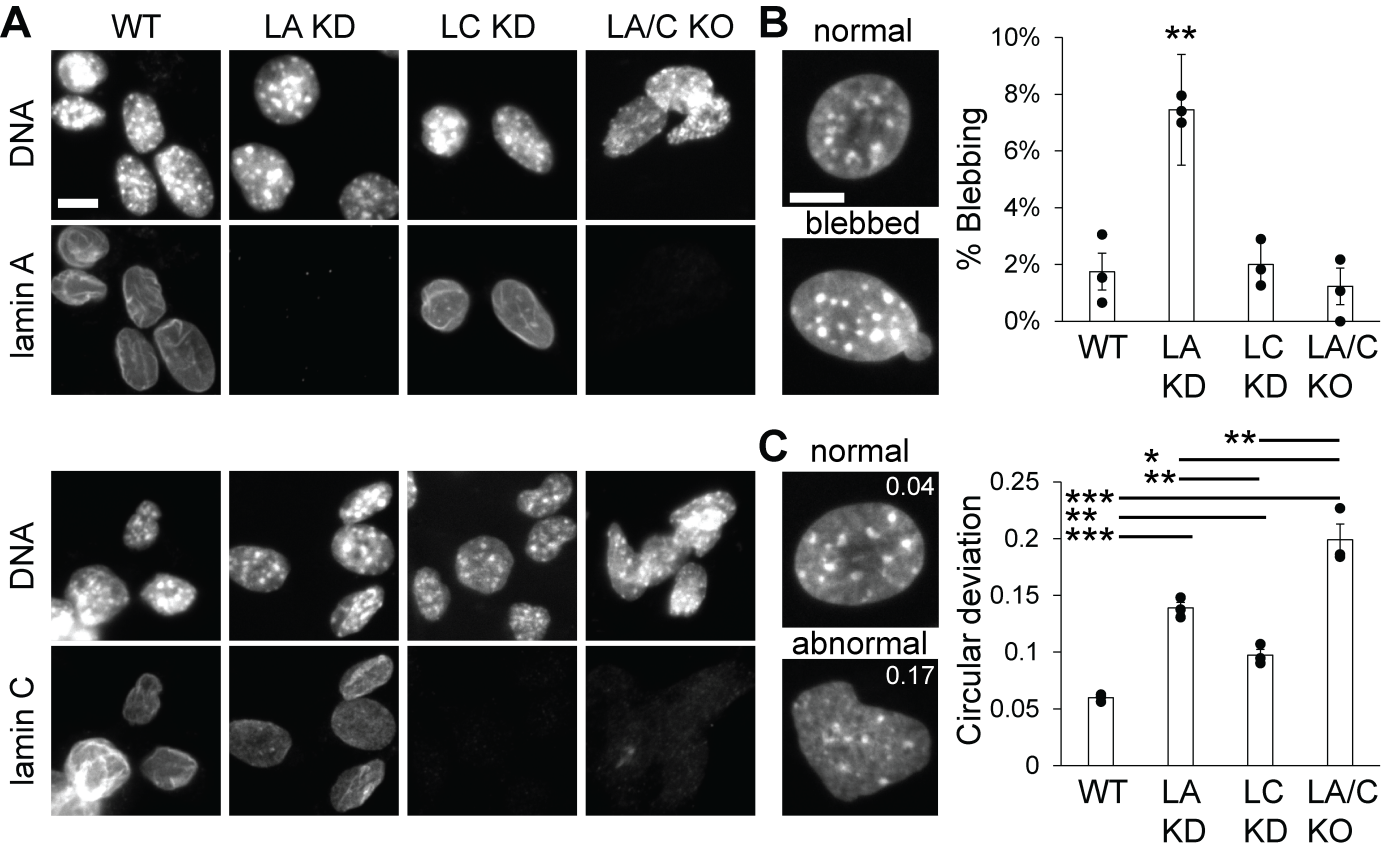
(A) Example images of wild type (WT), lamin A knockdown (LA KD), lamin C knockdown (LC KD), and lamin A/C knockout (LA/C KO) nuclei showing DNA (Hoechst) and lamin A and lamin C levels. (B) Representative images of normal versus blebbed nuclei. Graph of average percentage of blebbed nuclei in each condition. Three technical replicates consisting of WT 576, LA KD 323, LC KD 312, and LA/C KO 186 total cells. (C) Representative images of normal and abnormal nuclei with circular deviation values of 0.04 and 0.17, respectively. Graph of average circular deviation of nuclei in each condition. Three technical replicates consisting of WT 150, LA KD 138, LC KD 143, LA/C KO 120 total cells. Student’s t-test p values reported as * < 0.05, ** < 0.01, *** < 0.001, or no asterisk denotes no significance, p > 0.05. Error bars represent standard error. Scale bar = 10 µm.

## Description

Lamins, type V intermediate filaments, are major nuclear mechanical and structural contributors (Hobson et al., 2020; Lammerding et al., 2006; Pajerowski et al., 2007; Stephens et al., 2017; Swift et al., 2013; Vahabikashi et al., 2022) . Loss of nuclear shape leads to rupture which causes nuclear dysfunction that likely aids human disease via DNA damage, altering transcription, and disruption of cell cycle control (Chen et al., 2018; De Vos et al., 2011; Denais et al., 2016; Helfand et al., 2012; Irianto et al., 2016; Pfeifer et al., 2018; Raab et al., 2016; Shah et al., 2021; Stephens et al., 2019b; Xia et al., 2018) . Human afflictions presenting nuclear blebs and/or abnormal shape include aging, heart disease, and many cancers (Kalukula et al., 2022; Stephens et al., 2019a). The gene LMNA encodes for both lamin A and C making it difficult to separate their respective roles in the nucleus. A recent technological advancement generated stable cell lines that separately knockdown lamin A or lamin C via constitutive shRNAi (Vahabikashi et al., 2022; Wong et al., 2021) . Past studies reported that genetic knockout of LMNA results in abnormally shaped nuclei that deviate drastically from a circular shape (Broers et al., 2004; Chen et al., 2018; Lammerding et al., 2006; Nmezi et al., 2019; Robijns et al., 2016) . Therefore, we aimed to understand the separate roles of lamin A and lamin C in maintaining nuclear shape.

Immunofluorescence staining of lamin A and lamin C was first conducted to confirm that these stable cell lines were depleted of their respective lamins. Wild type mouse embryonic fibroblasts show clear staining for both lamin A and C while DNA dye Hoechst provides a secondary reporter for the nucleus ( Figure 1A ). Loss of lamin A and lamin C immunofluorescence occurred in their respective constitutively shRNAi expressing knockdown cell lines. Finally, genetic knockout of the LMNA gene shows a clear loss of both lamin A and C immunofluorescence.

Nuclear blebbing has been directly shown to cause nuclear rupture and dysfunction (Pho et al., 2024; Stephens, 2020; Stephens et al., 2019b). Thus, we measured the percentage of nuclei that displayed a nuclear bleb, a > 1 µm protrusion of the nucleus ( Figure 1B ) (Stephens et al., 2018) . Wild type MEFs display 2±1% nuclear blebbing. Knockdown of lamin A displayed a statistically significant increase in nuclear blebbing to 7±1%. However, knockdown of lamin C 2±1% or knockout of lamin A/C 1±1% did not significantly change nuclear blebbing relative to wild type. Thus, only loss of lamin A results in increased nuclear blebbing for MEF cells.

Loss of lamin A/C has been largely reported to cause abnormal nuclear shape that can be generally measured by deviation from a circle. Specifically, we measured circularity then graphed it as circular deviation which is 1-circularity where a perfect circle is 1 circularity or 0 for circular deviation ( Figure 1C ). Wild type cells have a low circular deviation at 0.06±0.01 and all lamin perturbations showed a statistically significant increase in circular deviation. Lamin C knockdown results in a modest but significant increase in circular deviation to 0.09±0.01. Next, lamin A knockdown resulted in a statistically greater level of increased circular deviation 0.14±0.01, though not surprising given the increase in nuclear blebbing. Finally, lamin A/C knockout resulted in the greatest statistically significantly increase in circular deviation compared to all other conditions at 0.20±0.01. Lamin A knockdown circular deviation largely stems from nuclear blebbing while lamin A/C knockout’s does not. Overall, taken together loss of lamin A, C, and A/C affect nuclear shape distinctly.

These findings show that loss of both lamin A/C in a LMNA knockout is significantly different than the loss of either lamin A or C. Many studies intermix lamin A, C, and A/C and their effects on nuclear shape, mechanics, and function. Our data clarifies that each of these perturbations results in a unique phenotype and outcome. Our work recapitulates a study showing that nuclear blebbing occurs upon lamin B depletion and dual lamin A and B depletion, but nuclear blebbing is lost upon depletion of all lamins (A, C, B1, B2) and instead presents an abnormal shape (Chen et al., 2018) . Our findings ( Figure 1 ) and this other published work both show that loss of lamin A or lamin B causes nuclear blebbing and, upon additional loss of lamin C, nuclear blebbing is lost and is replaced by abnormal shape. This finding is interesting considering that loss of lamin C itself only has a modest nuclear shape effect. Future work will be needed to test the hypothesis that nuclear lamin C presence is an essential player in nuclear bleb formation not previously investigated. In summary, many recent findings show clear differences between loss of lamin A, lamin C, and both lamin A/C.

## Methods

**Cell culture**

Mouse Embryonic Fibroblast (MEF) wild type (WT), lamin A constitutive knockdown via shRNAi (LA KD), lamin C constitutive knockdown via shRNAi (LC KD), and lamin A/C knockout (LMNA -/-, LA/C KO) cells were previously described (Berg et al., 2023; Shimi et al., 2008; Stephens et al., 2018; Vahabikashi et al., 2022; Wong et al., 2021) . Cells were cultured in DMEM (Corning) containing 10% fetal bovine serum (FBS, HyClone) and 1% penicillin/streptomycin (Corning). The cells were incubated at 37°C and 5% CO _2_ and passaged every 2-3 days. Cells were cultured for no longer than 30 generations. LA KD and LC KD cells were put under selection with 50 µg/mL G418 (Corning, 30-234-Cl) in complete DMEM throughout culturing.

**Immunofluorescence and fixed cell imaging**

As previously described in (Pho et al., 2024) , cells were grown in 8-well cover glass chambers (Cellvis) to 80% confluency. Cells were then fixed with 4% paraformaldehyde (Electron Microscopy Sciences) in PBS (Corning) at room temperature for 15 minutes. Cells were next washed with PBS 3 times, 5 minutes per wash. Following fixation, the cells were permeabilized with 0.1% Triton X-100 (US Biological) with PBS for 15 minutes at room temperature. The cells were then washed with 0.06% Tween 20 (US Biological) in PBS for 5 minutes, followed by 2 more washes in PBS, 5 minutes per wash. The cells were blocked with 10% goat serum (Sigma-Aldrich) in PBS for 1 hour at room temperature.

Primary and secondary antibodies were diluted with the blocking solution (10% goat serum in PBS, Sigma). The primary antibodies used were rabbit anti-Lamin A at 1:500 (#323 Goldman lab (Dechat et al., 2007) ) and rabbit polyclonal anti-Lamin C at 1:100 (ab125679, Abcam). Primary antibodies were added to the 8 well dish for 12 hours at 4°C. The cells were then washed with PBS 3 times, 5 minutes per wash. The secondary antibody used was Alexa Fluor 647 Anti-Rabbit IgG 1:1000 (4414, Cell Signaling Technologies), added to the dish and left to sit for 1 hour at room temperature. The cells were washed with PBS 3 times, 5 minutes per wash.

The cells were then stained with a 1 µg/mL dilution of Hoechst 33342 10mg/mL (H3570, Invitrogen) in PBS for 5 minutes before washing with PBS 3 times. Afterwards, the dish was mounted with ProLong Gold antifade (Life Technologies) and left to cure at room temperature for 12 hours.

**Microscope imaging and analysis**

Images were captured with Nikon Elements software on a Nikon Instruments Ti2-E microscope with Crest V3 Spinning Disk Confocal, Hamamatsu Orca Fusion Gen III camera, Lumencor Aura III light engine, TMC CleanBench air table, with 40x air objective (N.A 0.75, W.D. 0.66, MRH00401 and 16-bit camera. Immunofluorescence and live cell images were taken at 0.5 µm z-steps over 4.5 µm (9 steps). Lamin A and C loss was validate by presence or absence of immunofluorescence staining.

For live cell imaging for nuclear blebbing and shape, cells were grown to 80% confluency in 4-chamber glass bottom wells (Cellvis). Prior to imaging, cells were stained with a 1 µg/mL dilution of Hoechst 33342 (Life Technologies) for 15 minutes at 37°C, 5% CO _2_ . Images were taken via the Hamamatsu Orca Fusion camera 12-bit sensitive mode with 40x air objective N.A 0.75. Images were saved and analyzed in the NIS-Elements AR Analysis software. Images were observed to count the total number of nuclei and blebs in each field of view. ROIs were drawn around individual nuclei over Hoechst fluorescent images using the NIS-Elements threshold or by hand. Circularity measurements were then taken from the ROIs and exported from the NIS-Elements software to Excel for averages and statistical significance determined using the *t* test.

## Reagents

**Table:** 

**Reagent type**	**Name**	**Available from**
Antibody	Rabbit anti-Lamin A	#323 Goldman lab (Dechat et al *.* , 2007)
Antibody	Rabbit polyclonal anti-Lamin C	Abcam, ab125679
Antibody	Alexa Fluor 647 Anti-Rabbit IgG	Cell Signaling Technologies, 4414
DNA dye	Hoechst 33342	Invitrogen, H3570
Antibiotic	G418	Corning, 30-234-Cl
